# Finite Element Analysis of Grouting Compactness Monitoring in a Post-Tensioning Tendon Duct Using Piezoceramic Transducers

**DOI:** 10.3390/s17102239

**Published:** 2017-09-29

**Authors:** Tianyong Jiang, Junbo Zheng, Linsheng Huo, Gangbing Song

**Affiliations:** 1 School of Civil Engineering, Changsha University of Science and Technology, Changsha 410114, China; tianyongjiang@csust.edu.cn (T.J.); junbozheng@yeah.net (J.Z.); 2 School of Civil Engineering, Dalian University of Technology, Dalian 116024, China; lshuo@dlut.edu.cn; 3 Department of Mechanical Engineering, University of Houston, Houston, TX 77204, USA

**Keywords:** Lead Zirconate Titanate (PZT), smart aggregate (SA), post-tensioning tendon duct (PTTD), grouting compactness, grouting compactness monitoring, finite element model (FEM)

## Abstract

With the development of the post-tensioning technique, prestressed concrete structures have been widely used in civil engineering. To ensure the long-term effectiveness of the prestressed tendon, the grouting quality of the tendon duct is one of the important factors. However, it is still a challenge to monitor the grouting quality of post-tensioning tendon ducts, due to the invisibility of the grouting. The authors’ previous work proposed a real-time method that employed a stress wave-based active sensing approach with piezoceramic transducers to monitor the grouting compactness of a Post-Tensioning Tendon Duct (PTTD). To further understand the piezoceramic induced stress wave propagation in the PTTD with different grouting levels, this paper develops a two-dimensional finite element model for monitoring the grouting compactness of the tendon duct with a piezoceramic transducer. A smart aggregate (SA) developed to utilize one Lead Zirconate Titanate (PZT) transducer with marble protection is installed in the center location of the tendon duct as an actuator. Two PZT patches are bonded on the bottom and top surface of the tendon duct as the sensors. The analysis results show that the finite element analysis results are in good agreement with the experimental results, which demonstrates that the finite element analysis is feasible and reliable. For the top half of the specimen, not much stress wave could be detected before the full grouting level, except for negligible signals that may propagate through the walls of the tendon duct. When the tendon duct grouting is at 100%, the stress wave propagates to the top of the specimen, and the displacements are symmetric in both left-right and top-bottom directions before the stress waves reach the boundary. The proposed two-dimensional finite element model has the potential to be implemented to simulate the stress wave propagation principle for monitoring grouting compaction of the post-tensioning tendon duct.

## 1. Introduction

Grouting compactness of Post-Tensioning Tendon Ducts (PTTD) can influence the durability of and load-bearing capacity of post-tensioning concrete structures, and poor grouting compactness can even sometimes result in collapse of post-tensioning concrete structures. To guarantee durability and load-bearing capacity of the post-tensioning concrete structures, it is necessary to fully fill the post-tensioning tendon duct with grout to prevent inflow of air and moisture and to prevent corrosion of steel tendons. During a grouting process, due to blockage in the duct and improper grouting methods, there might be voids in the tendon ducts [1,2,3]. In consideration of the invisibility of post-tensioning tendon ducts in prestressed concrete structures, there are non-destructive evaluation (NDE) and structural health monitoring (SHM) methods for monitoring grouting quality or defects.

Ultrasonic imaging methods have been used for detection of tendon ducts. Krause et al. presented advantages using ultrasonic imaging for detecting grouting conditions in post-tensioning tendon ducts. Experimental results showed that ultrasonic imaging can accurately indicate the location of grouting faults in tendon ducts with fast ultrasonic measurement using an ultrasonic linear array [4,5,6,7]. Muldoon et al. verified the presence of voids in plastic ducts in post-tensioning concrete using ground-penetrating radar (GPR) [8]. However, a GPR cannot obtain the grouting condition within the steel ducts due to electromagnetic wave-shielding effect. Zou et al. used an impact-echo (IE) method to detect post-tensioning tendon ducts and evaluate its grouting defect position under various grouting conditions [9]. Abraham and Cote used the impact-echo method to monitor grouting quality of post-tensioning tendons based on a shift in the slab thickness resonant frequency above partially empty tendon ducts. Moreover, it was demonstrated that this method was effective for both metal and plastic ducts [10]. However, in general, the above methods are not suitable for real-time monitoring, Lead Zirconate Titanate (PZT) transducers, which have the advantages of low cost, wide bandwidth, strong piezoelectric effect, availability in different shapes, and sensing and actuating abilities, have been extensively researched for structural health monitoring of various structures [11,12,13,14,15,16,17]. To bridge the gap between laboratory studies and engineering practice, Lim et al. used PZT to perform a series of experimental studies on practical issues, such as the consistency of wave velocities, repeatability of the sensor’s electrical signatures, and the optimum frequency of actuation in concrete structures [18]. Lu et al. developed an electromechanical impedance method to monitor the strength of cementitious materials using the resonance frequency of a PZT-based smart probe [19]. Tian et al. used PZT transducers with a time-reversal method to monitor the grouting quality of post-tensioning tendon ducts. The grouting compactness can be obtained by evaluating the peak value changes of the time-reversal focused signal [20]. A PZT-based smart aggregate (SA) was developed as a transducer for monitoring concrete structural health conditions [21,22]. Jiang et al. adopted an SA and embedded it in the central position of the tendon duct as an actuator. Meanwhile, PZT patches were surface-bonded to the bottom and top surfaces of the tendon duct as sensors. The experiment results showed the wavelet packet energy of the PZT sensor could analyze the grouting compactness of the post-tensioning tendon duct [23]. To accelerate the applications of PZT-based methods for grouting quality monitoring in thepost-tensioning tendon ducts of concrete structures, it is necessary to establish a finite element model of the structural with the SA and the PZT patches involving piezoelectric and dielectric behavior for accurately predicting the grouting defects in the tendon ducts.

Numerical simulations of structures involving piezoelectric electric elements have been reported in the literature. Cappon et al. adopted the method of integrating numerical simulations and experimental data to obtain the valid piezoelectric material parameters [24]. Filoux et al. developed a hybrid algorithm combining finite-difference and pseudo-spectral methods to accurately simulate both the generation of acoustic waves by the piezoelectric transducer and their propagation in the surrounding media [25]. Piranda et al. developed a numerical model updating technique adapted to the piezoelectric transducer for accurately predicting the structure response [26]. Lahmer et al. proposed an enhanced iterative method for the precise reconstruction of piezoelectric material parameters based on finite element simulation and measurements of the electrical impedance [27]. Wolf et al. presented the method for the identification of further material parameters of a transducer assembly [28]. Joo et al. proposed an inversion method for obtaining the impedance of piezoelectric transducers by using a three-dimensional finite element [29]. Her and Lin used the commercial software ANSYS based on three-dimensional finite elements to simulate the active vibration control of smart structural systems using piezoelectric materials as actuators. The results showed that the numerical results were in good agreement with the theoretical prediction [30]. Kusculuoglu et al. presented a finite element model of a beam with a piezoceramic patch, and the model was able to simulate both active and passive control [31]. Providakis et al. investigated the finite element models of damages occurring in conventional unreinforced, steel-reinforced or fiber reinforced plastics-reinforced concrete specimens [32]. Zhang et al. designed and simulated a circular piezoelectric unimorph with the edge clamped for the use as an ultrasound transducer. The numerical results showed that the optimized dimension of the PZT layer could improve the efficiency of the transducer [33]. Liang et al. performed finite element model analysis to simulate the debonding behavior of a concrete-steel composite structure. The numerical impedance signature results agreed well with the experimental results, which validates the electro-mechanical impedance (EMI) technique for detecting the debonding behavior of concrete-steel composite structures [34]. Liang et al. presented a surface-bonded piezoelectric 3D finite element method to simulate and analyze the impedance signature of a pin connection model [35]. Luo et al. used a finite-difference numerical method to simulate the concrete infill monitoring in concrete-filled FRP tubes [36]. The experimental results showed good agreement with the numerical findings, which validates the ultrasonic time-of-flight method.

The authors’ previous work proposed a stress-wave-based active sensing approach using piezoceramic transducers to monitor the grouting compactness of PTTD in real time [23]. To further understand the stress wave propagation in a PTTD with different grout levels, in this paper, the authors develop a finite element numerical model for a PTTD with piezoceramic transducers based on the transient analysis method. In the model, a smart aggregate (SA) employing one Lead Zirconate Titanate (PZT) piezoceramic transducer with marble protection is installed in the center location of the tendon duct as an actuator. Two PZT patches are bonded to the bottom and top surfaces of the tendon duct as the sensors. Piezoelectric and dielectric behaviors of the SA and PZTs in the finite element model are provided. The SA actuator excites the sinusoidal signals with a voltage of 10 V and a fixed frequency of 80 kHz. The signal response of the PZT sensors of the tendon duct is collected. The experiments validate the numerical analysis under three different grouting conditions, such as 50% grouting, 90% grouting, and 100% grouting. The displacement contour diagram shows the complete wave propagation path in the post-tensioning tendon duct. 

## 2. Finite Element Model

**Figure 1:**
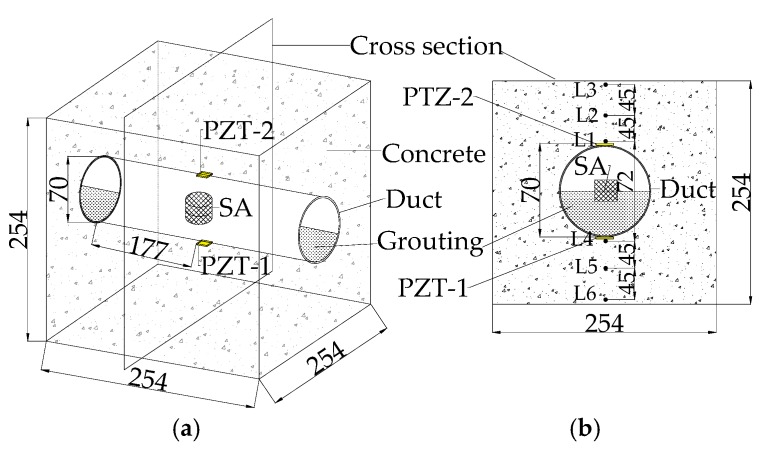
The geometric details of the grouting compactness monitoring model (unit: mm). (**a**) 3-dimensional view of the test specimen. (**b**) Sectional view of the specimen.

A finite element model was established to analyze the propagation principle and monitor the grouting compactness in post-tensioning tendon ducts using an active sensing approach based on piezoceramic transducers. Commercial software, ANSYS, was used to develop the grouting compactness monitoring numerical model. The geometric details of the grouting compactness monitoring model are shown in Figure 1. 

Two PZT patch sensors, including one sensor (PZT 1) bonded to the bottom outside surface and the other sensor (PZT 2) mounted on the top outside surface of the tendon duct, were used as sensors to receive stress waves. One SA, embedded in the center location of the tendon duct, was employed as an actuator to excite the sinusoidal signals with voltage of 10 V and a fixed frequency of 80 kHz. The SA is a cylinder with diameter of 25 mm and height of 25 mm. The concrete specimen is a cube, with each side having a length of 254 mm. The outer diameter of the tendon duct is 70 mm, and its thickness is 5 mm. A two-dimensional numerical model was established along the cross-section through the central location of specimen, as shown in Figure 1b. The ANSYS two-dimensional model involved the concrete specimen, including the duct, the grouting, PZT patches and the SA. The type of PZT transducer used for the PZT sensors and SA in the numerical model was a PZT-5H, with dimensions of 15 mm × 0.3 mm. 

**Figure 2:**
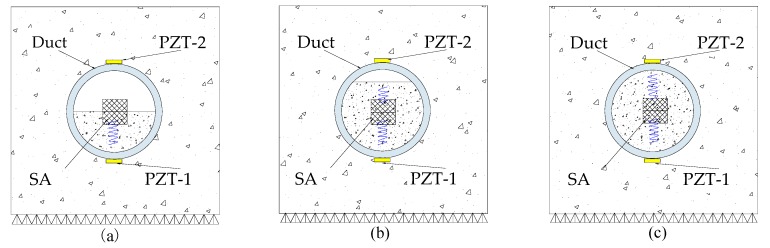
Three different grouting levels in tendon duct: (**a**) 50% grouting; (**b**) 90% grouting; (**c**) 100% grouting.

When the tendon duct is empty, there is no grout to allow the stress wave induced by the SA to travel and reach either one of the PZT sensors. To simulate the grouting progress of the post-tensioning tendon duct, three different grout levels are involved—50%, 90%, and 100%—as shown in Figure 2. When the grout level is 50%, as shown in Figure 2a, the bottom PZT sensor can receive the stress waves induced by the SA, however the top PZT sensor still cannot receive much signal. For the top half of the specimen, not much stress wave can be detected before the full grouting level, as shown in Figure 2b, except for negligible signals that may propagate through the walls of the tendon duct. At the 100% grouting level, the stress wave can reach both the bottom and the top PZT transducers, as shown in Figure 2c.

The material properties of the concrete, grouting, duct and marble in the ANSYS finite element model are presented in Table 1. A PZT transducer model requires an elastic coefficient matrix $C^{E}$, piezoelectric stress constant matrix e and dielectric permittivity matrix $\varepsilon^{s}$ as material properties. The PZT-5H material is polarized in the *Y* direction in the ANSYS finite element model. The elastic coefficient matrix $C^{E}$ is expressed in the form

(1) $$\left[C^{E}\right]=\left[\begin{array}{cccccc} C_{11} & C_{13} & C_{12} & 0 & 0 & 0\\ C_{13} & C_{33} & C_{13} & 0 & 0 & 0\\ C_{12} & C_{13} & C_{11} & 0 & 0 & 0\\ 0 & 0 & 0 & C_{44} & 0 & 0\\ 0 & 0 & 0 & 0 & C_{44} & 0\\ 0 & 0 & 0 & 0 & 0 & C_{66} \end{array}\right]=\left[\begin{array}{cccccc} 13.9 & 7.43 & 7.78 & 0 & 0 & 0\\ 7.43 & 1.15 & 7.43 & 0 & 0 & 0\\ 7.78 & 7.43 & 13.9 & 0 & 0 & 0\\ 0 & 0 & 0 & 2.56 & 0 & 0\\ 0 & 0 & 0 & 0 & 2.56 & 0\\ 0 & 0 & 0 & 0 & 0 & 3.06 \end{array}\right]\times 10^{10}(N/m^{2})$$

The dielectric permittivity matrix $\varepsilon^{s}$ is

(2) $$\left[\varepsilon^{s}\right]\;=\varepsilon_{0}\;\left[\begin{array}{ccc} \varepsilon_{r11} & & \\ & \varepsilon_{r33} & \\ & & \varepsilon_{r11} \end{array}\right]=8.85\times \left[\begin{array}{ccc} 919 & & \\ & 826 & \\ & & 919 \end{array}\right]\times 10^{12}(F/m)$$

where $\varepsilon_{0}$ is the free-space dielectric permittivity. 

The piezoelectric stress constant matrix e is:

(3) $$\left[e\right]=\left[\begin{array}{ccc} 0 & e_{31} & 0\\ 0 & e_{33} & 0\\ 0 & e_{31} & 0\\ e_{15} & 0 & 0\\ 0 & 0 & e_{15}\\ 0 & 0 & 0 \end{array}\right]=\left[\begin{array}{ccc} 0 & -5.2 & 0\\ 0 & 15.7 & 0\\ 0 & -5.2 & 0\\ 12.7 & 0 & 0\\ 0 & 0 & 12.7\\ 0 & 0 & 0 \end{array}\right](C/m^{2})$$

**Figure 3:**
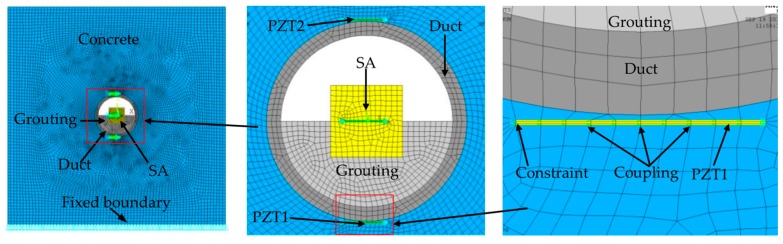
The mesh details of the finite element model. The blue is concrete, the light gray is grouting, the gray is duct, the yellow is PZT and SA, the green is coupling and the skyblue is constraint.

The PZT-5H transducer adopts a PLANE13 element, the tendon duct uses a PLANE223 element, and the concrete, the marble and the grout employ a PLANE182 element. The PZT transducer is bonded to the outer surface of the tendon duct using a thin layer of epoxy. The numerical model ignores the thin layer of epoxy. The mesh size of the outside edge of concrete is 2 mm, while the mesh size of the inside edge of the concrete is 1.5 mm. The mesh size of the duct, the marble of SA and the grout is 1.5 mm. The mesh size of the PZT transducer in the length direction is 1.5 mm, while in the width direction it is 0.1 mm. The mesh details of the finite element model are shown in Figure 3. In the Figure 3, the blue is concrete, the light gray is grouting, the gray is duct, the yellow is PZT and SA, the green is coupling, the skyblue is constraint. The *X* direction of the width edge of the PZT transducer is applied to the lateral constraint. The *Y* direction of bottom edge of the concrete is applied to the vertical displacement constraint. The top and bottom of the length edge of the PZT transducer are coupled as a whole in voltage freedom. And the bottom voltage of the length edge of the PZT transducer is set to zero voltage.

## 3. Experimental Verification

**Figure 4:**
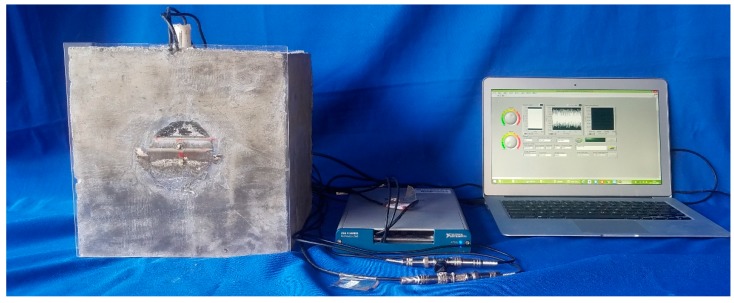
The experimental setup.

**Figure 5:**
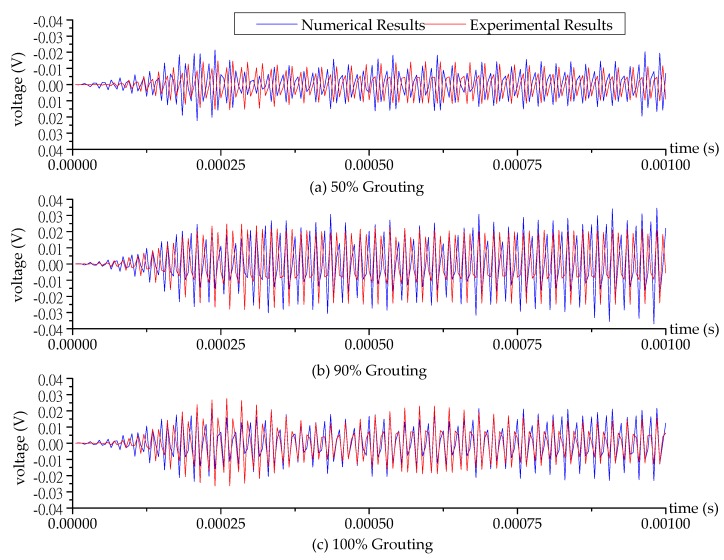
The numerical and experimental time-domain signal of the PZT 1 sensor at different grouting levels.

**Figure 6:**
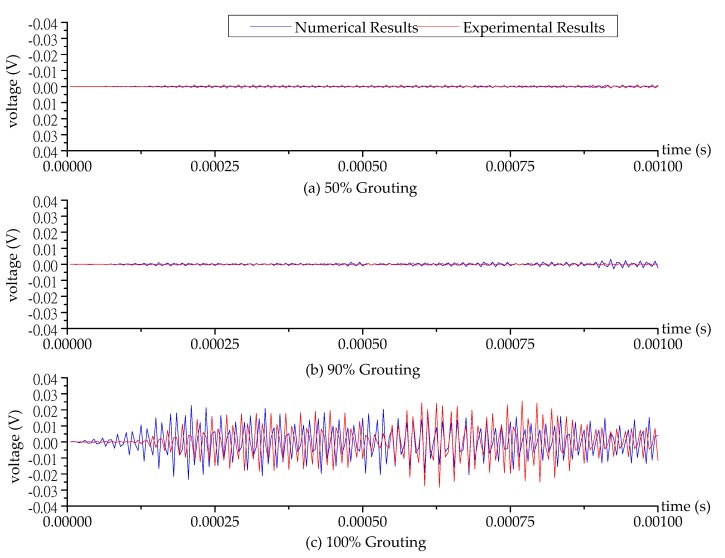
The numerical and experimental time-domain signal of the PZT 2 sensor at different grouting levels.

The schematic of the test specimen is shown in Figure 1. One SA was installed in the tendon duct. Two waterproofed PZT patch sensors were mounted on the outside surface of the tendon duct, one sensor (PZT 1) on the bottom and the other sensor (PZT 2) on the top, in the test specimen. The experimental setup included the test specimen, a data acquisition system (NI-USB 6331), and a supporting laptop, as shown in Figure 4. The data acquisition board was used to generate the signal to the SA and collect the stress wave detected by the PZT sensors. During the monitoring process, a fixed-frequency sine wave signal was used as the excitation signal to the SA actuator. Meanwhile, the signal response of each mounted PZT sensor was recorded. The frequency of the sine wave is 80 kHz. The amplitude of the swept sine wave is 10 V. One period of the signals is 0.0000125 s, therefore the 80 periods of the sinusoidal signals are 0.001 s, as shown in Figure 5 and Figure 6. 

When the grouting process reached 50%, 90%, and 100% grouting levels, the SA was excited by the sine signal and the responses of the PZT 1 sensor and PZT 2 sensor were recorded. Then, the data collected from the experimental specimen were processed with a bandpass filter employing a frequency range of 77 kHz to 83 kHz to remove noise and unrelated signals. The corresponding numerical and experimental time-domain signals received by the PZT 1 sensor and PZT 2 sensor at different grouting levels are shown in Figure 5 and Figure 6. Each figure reflects the sensor signal response, representing three critical grouting levels during the pouring process: (1) 50% grouting (Figure 5a and Figure 6a); (2) 90% grouting (Figure 5b and Figure 6b); and (3) 100% grouting (Figure 5c and Figure 6c). When the grout level reach to 50%, the grout between the SA and PZT 1 sensor allows the stress wave to propagate so that the PZT 1 sensor can receive sufficient signals at the 50% grouting level, and the received signals of PZT 1 sensor remain strong in the two following stages of 90% and 100% grouting levels, as shown in Figure 5. In addition, the signal received by PZT 1 sensor with 90% grouting level appears to be stronger than the 100% grouting level. When the grouting level is at 90%, the grout in the tendon duct is not full, and the stress waves reflected from the grout with the free boundary strengthen the signal of PZT 1 sensor. For the PZT 2 sensor bonded on the top surface of the tendon duct, not much signal can be obtained before the 100% grouting level, as shown in Figure 6. Only when the grout fully fills in the tendon duct, can the stress wave propagate to the top side of the tendon duct, and the PZT 2 sensor is then able to receive significant signals from the SA. 

In Figure 5, the results show that average numerical amplitude of the voltage signal of PZT 1 sensor is 0.0082 V, 0.0154 V, and 0.0108 V at 50%, 90% and 100% grouting level, respectively. While average experimental amplitude of the voltage signal of PZT 1 sensor is 0.0067 V, 0.0129 V, and 0.0101 V at 50%, 90% and 100% grouting level, respectively. From this, it can be seen that the numerical and experimental results are in good agreement. However, in Figure 6, we can see that the time when the PZT 2 sensor starts to receive the signal in the numerical model is earlier than that in the experimental one. Several factors, such as fabrication errors and inaccurate velocity of the stress wave in the concrete, may contribute this difference.

In summary, the finite element analysis results are in good agreement with the experimental results, which further demonstrated the feasibility and reliability of the finite element analysis. The two-dimensional finite element model can analyze the wave propagation to monitor and evaluate the grouting compactness of the post-tensioning tendon duct.

## 4. Further Numerical Analyses

**Figure 7:**
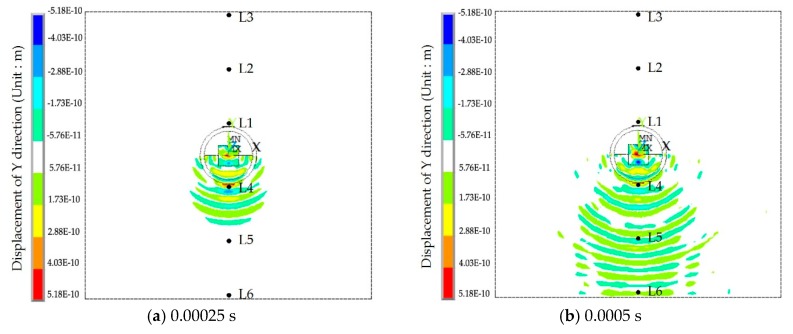

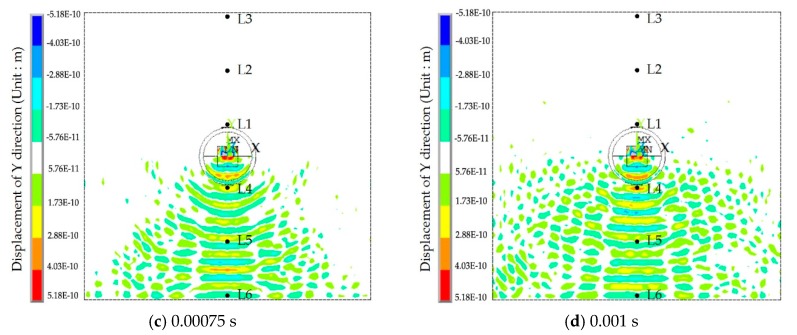
The displacement contour at the 50% grouting level.

**Figure 8:**
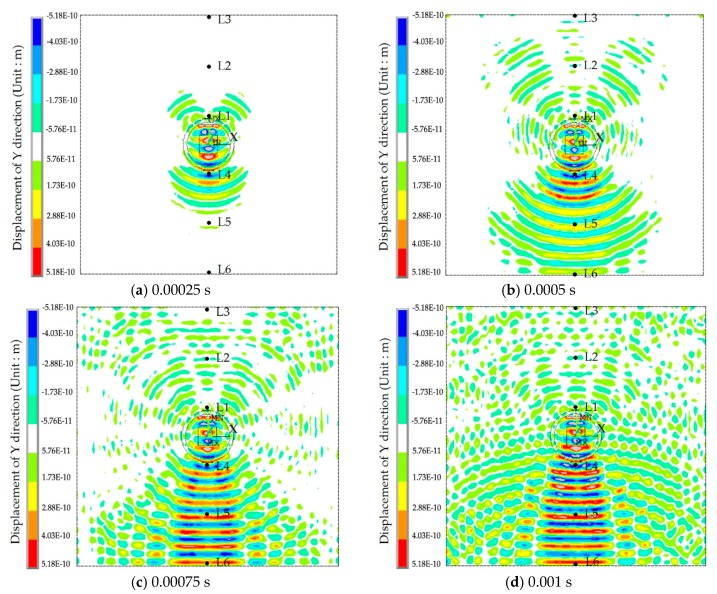
The displacement contour diagram at the 90% grouting level.

**Figure 9:**
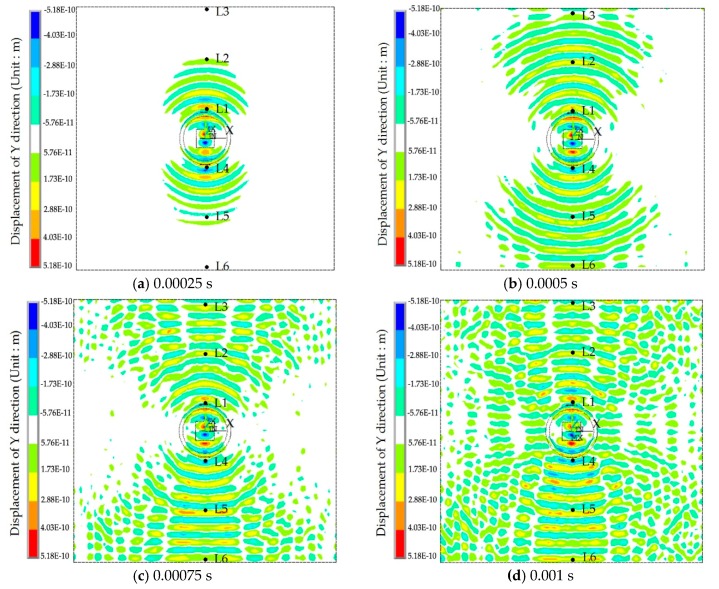
The displacement contour diagram at the 100% grouting level.

The displacement contour diagrams of the numerical model at 50% grouting level, 90% grouting level and 100% grouting level during stress wave propagation are shown in Figure 7, Figure 8 and Figure 9. Each figure reflects the displacement response of the numerical model when the SA excites the sinusoidal signals with the fixed frequency of 80 kHz, and represents four different time points during the signal propagation process: (1) 20 cycles, 0.00025 s (Figure 7a, Figure 8a and Figure 9a); (2) 40 cycles, 0.0005 s (Figure 7b, Figure 8b and Figure 9b); (3) 60 cycles, 0.00075 s (Figure 7c, Figure 8c and Figure 9c); (1) 80 cycles, 0.001 s (Figure 7d, Figure 8d and Figure 9d).

It can be seen that the displacement contour diagrams excited by the SA are left and right symmetrical. When the grouting level is 50% and 90%, the tendon duct contains a defect, and it is clear that the stress wave cannot propagate to the top half of the specimen, where only diffraction signals exist, as shown in Figure 7 and Figure 8. In Figure 8, the bottom half of concrete at the 90% level has a significant signal increase when the stress wave is reflected from the bottom of the concrete with the fixed boundary. 

At the 100% grouting level, the concrete specimen is structurally symmetric. When the tendon duct is 100% grouted, the displacement contour diagrams are symmetric in both left-right and top-bottom directions before stress waves reach the boundary, as shown in Figure 9. After stress waves arrive at the boundary, there are small differences between the top half and the bottom half of the concrete in the displacement contour diagrams, which display minor asymmetric behavior. This is mainly due to the different boundary conditions for the top and bottom surfaces of the specimen. The bottom of concrete has the fixed boundary and the top of concrete has the free boundary. The signal near the bottom of concrete will be strengthened by the reflected wave on the fixed boundary condition, while the signal near the top of concrete will be weakened by the reflected wave on the free boundary condition. When the sinusoidal waves execute 20 cycles (0.00025 s) in the numerical model, the stress waves pass through the grouting and tendon duct, and propagate in the concrete, but do not reach the edge of the concrete, as can be seen from Figure 7a, Figure 8a and Figure 9a. When the sinusoidal waves execute 40 cycles (0.0005 s), 60 cycles (0.00075 s), and 80 cycles (0.001 s) in the numerical model, the stress waves can propagate to a farther distance, so that they are able to reach the edge of the concrete, as shown in Figure 7, Figure 8 and Figure 9. And as the number of cycles of the sinusoidal waves execution increases, the distance the signals travel further.

**Figure 10:**
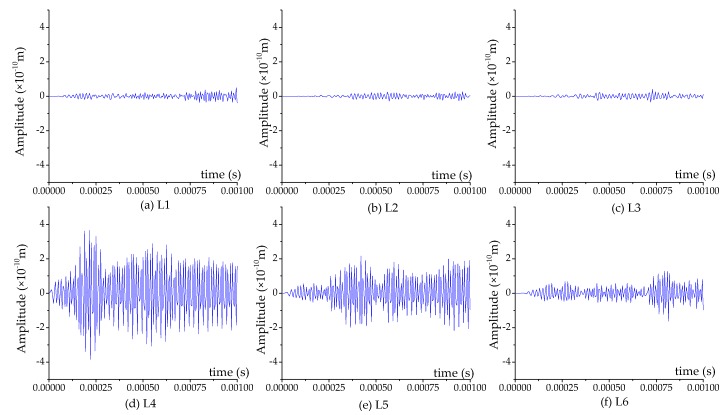
The displacement response at different locations at 50% grouting level.

**Figure 11:**
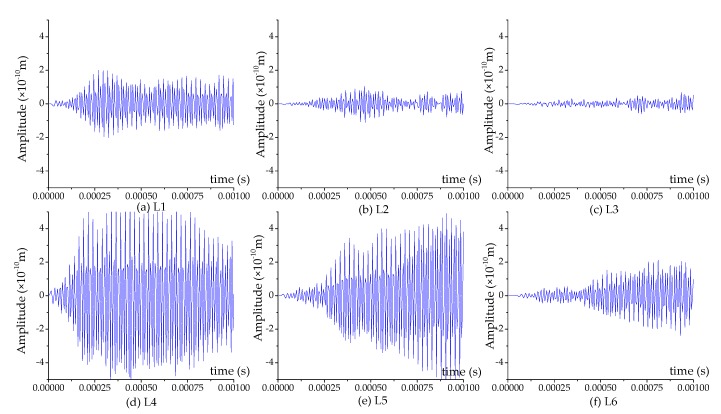
The displacement response at different locations at 90% grouting level.

**Figure 12:**
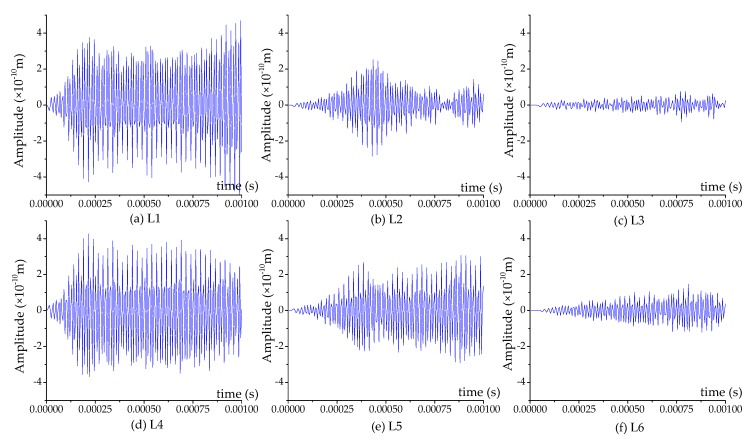
The displacement response at different locations at 100% grouting level.

Figure 10, Figure 11 and Figure 12 illustrate the displacement response at different locations of the specimen at 50%, 90% and 100% grouting levels, respectively. The different locations of the specimen include L1, L2, L3, L4, L5 and L6. Their location details are shown in Figure 1. L1, L2 and L3 are located in the top half of concrete structure, and are in turn on the inside edge, in the middle and on the outside edge. L4, L5 and L6 are located in the bottom half of concrete, and are also, in turn, on the inside edge, in the middle and on the outside edge.

For the top half and bottom half of the concrete, the displacement of the inside edge is the maximum, the middle is the second, and the outside edge is the minimum, as shown in Figure 10, Figure 11 and Figure 12. When the pouring progress achieves the 50% grouting level, the top half of the concrete only receives a weak signal, while the bottom half of the concrete receives a strong signal, as shown in Figure 10. When the grouting level reaches 90%, the top half of the concrete receives a signal that is stronger than that for the 50% grouting level; however, it is still weaker than that of the bottom half, as shown in Figure 11. Additionally, the signal intensity of the inner edge increases the most, the second in the middle and the least on the outside edge. When the grout fully fills in the tendon duct, the stress wave can directly arrive the top half of the concrete through the grout, hence the top half of concrete is able to receive significant signal from the SA, as shown in Figure 12. From Figure 10, Figure 11 and Figure 12, it can be seen that the response amplitudes of the outside edge of the concrete are not enough, but are relatively small at different grouting levels. This is mainly due to the fact that the outside edge of concrete is far from the SA, and the stress wave finds it difficult to reach it. Therefore, it is difficult to evaluate the grout compactness of the tendon duct by comparing the signals received by PZT sensors mounted on the outside edge of concrete. 

In summary, the two-dimensional finite element model can simulate the stress wave propagation principle for monitoring the grouting compaction of the post-tensioning tendon duct. In the authors’ future work, a three-dimensional finite element model will be established to study PZT transducer-based grouting compactness monitoring. In addition, the mobile based remote technology [37,38] will be integrated with proposed method for field implementation. 

## 5. Conclusions

The monitoring of the grouting compactness of a post-tensioning tendon duct (PTTD) is a difficult problem for post-tensioning concrete structures in civil engineering. The authors’ previous work proposed a real-time stress wave-based active sensing approach with piezoceramic transducers for monitoring the grouting compactness of a post-tensioning tendon duct. To further understand the wave prorogation in a PTTD with different grouting levels, this paper establishes a two-dimensional finite element model with piezoceramic transducers. The model was built along the cross section through the central location of a specimen that included the concrete structure, the duct, the grout, the PZT sensors and the SA. The SA was used as an actuator to excite sine signals with a fixed frequency of 80 kHz at the center location of the tendon duct. The PZT sensors mounted on the top and bottom surfaces of the tendon duct were utilized to receive the signal response. Similar results can be obtained from the numerical time-domain signals and the experimental time-domain signals at different levels. The numerical analyses show that, when the grouting level is 50% and 90%, the stress wave cannot directly propagate to the top half of the specimen. When the tendon duct has 100% grouting, the stress wave is able to propagate to the top of the specimen and can be detected by the PZT sensor mounted on the top of the duct. In summary, the two-dimensional finite element model is able to simulate stress wave propagation for monitoring the grouting compactness of the post-tensioning tendon duct. 

## Figures and Tables

**Table 1:** Material Properties [35,36].

Materials	Parameters	Values	Unit
Concrete	Density	2400	kg·m^3^
	Young’s modulus	32.5	GPa
	Poisson ratio	0.2	
Grouting	Density	2000	kg·m^3^
	Young’s modulus	15.8	GPa
	Poisson ratio	0.18	
Duct	Density	1380	kg·m^3^
	Young’s modulus	2.7	GPa
	Poisson ratio	0.38	
Marble	Density	2800	kg·m^3^
	Young’s modulus	50	GPa
	Poisson ratio	0.25	
